# Perceptions of Behaviors Associated with ASD in Others: Knowledge of the Diagnosis Increases Empathy and Improves Perceptions of Warmth and Competence

**DOI:** 10.3390/ejihpe12110112

**Published:** 2022-11-04

**Authors:** Deven L. Nestorowich, Shannon P. Lupien, Vicki Madaus Knapp

**Affiliations:** 1 Department of Psychological Sciences, Daemen University, 4380 Main St., Amherst, NY 14226, USA; 2 Department of Behavioral Science, Daemen University, 4380 Main St., Amherst, NY 14226, USA

**Keywords:** autism spectrum disorder, warmth, competence, empathy, cost of cognition

## Abstract

Individuals with Autism Spectrum Disorder (ASD) often exhibit atypical social behaviors that some may perceive as odd or discomforting. Given that ASD is largely invisible, it may be difficult to understand why a person is displaying these atypical behaviors, leading to less favorable attitudes. The current study aimed to examine if having an explanation for an individual exhibiting behaviors associated with ASD could improve perceptions of warmth and competence, as well as the amount of empathy felt towards the individual. Participants (*n* = 82) were presented with a scenario involving two people, one of whom exhibited behaviors consistent with ASD. ASD diagnosis information was manipulated, such that half of the participants were told that the target was diagnosed with ASD, and the other half were given no diagnostic information. Afterwards, participants rated the target. Results indicated that having an explanation for the ASD-related behaviors led to higher ratings of warmth and competence and greater feelings of empathy. Furthermore, empathy mediated the relationship between having the diagnostic information and target ratings. Thus, having an explanation for someone’s behavior may lead to greater feelings of empathy and improve perceptions and understanding. This has important implications for improving education and awareness about behaviors associated with ASD as well as for making the decision of whether or not to disclose one’s diagnosis.

## 1. Introduction

In the words of esteemed psychiatrist and psychoanalyst Carl Jung, “If one does not understand a person, one tends to regard him as a fool,” [1]. This sentiment suggests that we need to be able to understand others in order to empathize with them and perceive them more positively. This likely poses difficulties for individuals who have developmental or social differences, such as Autism Spectrum Disorders (ASD), who tend to exhibit atypical or unfamiliar social behaviors that some may not understand or perceive as odd or discomforting. Research indicates that there is an overall large decrease in normative social conduct between those with ASD and those without [2,3]. According to the DSM-5-TR, ASD is defined by differences in social behaviors, such as “deficits in social-emotional reciprocity...in nonverbal communication…” and “in developing, maintaining, and understanding relationships,” [4]. These traits manifest themselves in the lack of understanding of subtext, an inability to understand the emotional state of others, and ignorance of social etiquette for novel situations. Indeed, research has indicated that there are certain stereotypes associated with ASD, including having poor social skills, being withdrawn, having poor communication, and having a difficult personality or engaging in difficult behavior, all of which are perceived as negative traits [5]. Additionally, individuals who have been diagnosed with ASD are often stigmatized [6,7] and experience bullying at a disproportionate rate compared to their peers [8]. They may also experience discrimination that has important implications for their success. For example, it was found that the tendencies for those with ASD to have sensitivity to sensory input and difficulties when unable to adhere to a routine were found as problematic qualities for the work environment and seen as reasons why they would make for poor employees [9]. Thus, it is important to investigate ways to improve perceptions among individuals displaying behaviors associated with ASD.

The current study explores whether giving individuals diagnostic information about a person exhibiting behaviors associated with ASD can increase empathy and thereby improve perceptions, particularly on dimensions of social competence and warmth, which are two primary dimensions of social perception on which humans are evolutionarily predisposed to judge others and have been shown to form the basis of over 80% of people’s impressions of others [10,11,12,13]. Furthermore, the dimensions of warmth and competence have been argued to underlie stereotypes. In fact, this is a primary principle of the stereotype content model (SCM), which indicates that although specific beliefs about particular groups of people may contribute to stereotypes—like the ones mentioned above related to behaviors associated with ASD—the majority of the variance in stereotypes stem from these fundamental dimensions of warmth and competence [10]. Thus, if perceptions of warmth and competence can be improved for someone exhibiting behaviors associated with ASD, it may help to decrease the associated negative stereotypes.

Several studies support the idea that knowledge of or experience with ASD, or awareness of an ASD diagnosis may lessen the stigma often associated with the disorder [6,7,9,14,15,16,17]. First, greater knowledge of ASD has been shown to predict decreased stereotyping and thereby reduced social distancing from individuals diagnosed with ASD [18]. Additionally, when given a vignette that included a diagnosis label of ASD compared to a vignette with behaviors associated with ASD, participants were more likely to stigmatize the behaviors, but not the label [19]. This demonstrates that although behaviors associated with ASD may indeed be associated with stigma, having a label of ASD diagnosis may be less so. Thus, it is important to focus on perceptions of *behaviors* associated with ASD and how knowledge of ASD or diagnostic information may actually help to improve perceptions. Specifically, participants more knowledgeable about ASD were more likely to recommend hiring candidates with a disclosed ASD diagnosis compared to candidates without a disclosed diagnosis. However, this only occurred when the job candidates did not have observable characteristics associated with ASD [9]. Therefore, previous knowledge of ASD may only improve perceptions when a person is exhibiting relatively normative social behavior. A more concrete explanation, such as diagnostic information, may be required for non-normative behavior that is often associated with ASD. One study demonstrated this among teachers and their perceptions of students. Participants who read vignettes depicting common behaviors among primary-school students diagnosed with ASD, rated the behaviors more positively when they were made aware of the students’ ASD diagnoses compared to when they were unaware of the diagnoses [16]. Another study indicated that participants’ affective responses were more positive after reading about a target displaying behaviors associated with ASD when the target was presented as someone with an ASD diagnosis compared to a typical college student [14]. Similarly, Matthews and colleagues (2015) presented vignettes of individuals exhibiting behaviors associated with ASD and found that a diagnosis of ASD at Level 1, requiring some (not substantial) support, resulted in more positive behavioral and cognitive responses compared to no diagnostic information [15]. Thus, it is clear from previous research that providing participants with diagnostic information about a person exhibiting behaviors associated with ASD may result in more positive responses. One of the aims of the current study is to extend these findings to the primary dimensions of warmth and competence that have been shown to underlie stereotypes [10].

There may be several reasons why providing diagnostic information may improve responses towards those exhibiting behaviors associated with ASD. One reason is that ASD is largely invisible, and people who have an ASD diagnosis do not look any different from an average person, but they may often engage in non-normative social behavior [2,3,4]. When individuals encounter these behaviors, they may become uncomfortable or apprehensive because there is no explanation for why someone may be exhibiting such behavior, which likely requires more cognitive effort to understand. However, these same behaviors are often exhibited in autism-coded characters in the media but are perceived more favorably. Autism coding is a term used to describe characters in media that appear to have behaviors similar to those exhibited by individuals with ASD. For example, autism coding is often applied to non-human entities to contrast with their human counterparts. As such, ASD-like traits are used to differentiate these entities from humans as a way to show they are not really human despite apparent similarities. Popular and often beloved examples include Janet from The Good Place, Data from Star Trek, Drax from Guardians of the Galaxy, and Vision from the Marvel Cinematic Universe. They all look very similar to humans but are often differentiated by displaying atypical or unexpected social behaviors often associated with ASD. Autism-coded characters exhibiting these behaviors are typically seen as charming and endearing. Unlike their autism-coded counterparts, these same behaviors expressed in humans are often seen more negatively [5]. There may be one major difference between these two groups that may explain why one is perceived as charming and the other more negatively. In autism-coded characters, the audience is given explicit reasons as to why they behave the way they do—they are non-human. Those with ASD in real life do not have the luxury of a built-in explanation for their behaviors. Therefore, it is possible that if someone knew that another had ASD and what that entailed, it would provide a clear explanation, making the behaviors seem more familiar and perhaps result in more favorable evaluations.

Research indicates that people find it easier to perceive and cognitively process familiar stimuli, which results in more “fluent,” processing—understanding information more easily with less cognitive effort required. Additionally, because people find the experience of fluency inherently pleasurable, those positive feelings make the stimuli more appealing [20,21]. As such, people may be positively reinforced by this desired experience of fluency, and thus encountering unfamiliar stimuli may lead to less favorable judgments of the given stimuli. The subjective experience of fluency influences all sorts of judgments people are called on to make [22,23]. Therefore, encountering individuals with ASD engaging in unexpected social behaviors could also potentially lead to a decreased sense of fluency and, in turn, less favorable judgments. Studies have found that people are significantly less likely to empathize with someone when doing so would be more cognitively taxing. Participants empathized with a scenario more if it was high efficacy (clear) and required little cognitive effort, compared to if it was low efficacy (vague) and required a good deal of thought [19]. This is important because having high levels of cognitive empathy has been linked with having less stigma towards those with ASD [24]. Since those with ASD may display unexpected behaviors that are more difficult to understand, social interactions with them may be considered low efficacy and thus more cognitively taxing. Therefore, another aim of the current study is to investigate if providing diagnostic information about someone exhibiting behaviors associated with ASD may increase empathy, presumably by increasing the efficacy of the situation, making it less cognitively taxing by providing an explanation for the atypical behavior.

Furthermore, this increased empathy may relate to dimensions of warmth and competence, because according to the SCM, higher perceptions of warmth and competence are often felt towards one’s ingroup [10,25], and it has been argued that empathy—taking on the perspective of others—can lead people to perceive less differences between themselves and out-group members (i.e., empathy may increase perceptions that others are part of one’s ingroup, thereby increasing perceptions of warmth and competence) [26]. Supporting this idea, research has shown that people are more likely to dehumanize outgroup members [27] and that dehumanization has been associated with certain mental disorders, including ASD. Furthermore, dehumanization has been negatively correlated with competence and warmth [25].

Additionally, people have been shown to associate typical human behavior with ingroup members as opposed to out-group members [28], which may suggest that the atypical behaviors associated with ASD may lead to an outgroup categorization and therefore lower feelings of empathy and lower perceptions of warmth and competence. For example, it was found that many of the same perceived social deficits of those with ASD were found in social robots, namely issues with body language and maintaining eye contact. Results from one study revealed that while most adults felt uneasy with a social robot’s body language, children with ASD did not [29]. The unease the adults felt seemed to stem from a lack of familiarity with the behaviors. The children however, likely exhibited similar behaviors themselves, and thus it is possible that because they were more familiar with those behaviors, they were more likely to categorize them as part of their ingroup, leading to more positive perceptions. Similarly, another study found that while the majority of children aged 15 categorized a social robot as being different from them in social, mental, and moral attributes, a majority of children aged 9 and 12 did not [30]. This suggests that the sense of otherness being associated with different social behavior may be a learned trait, rather than one that is naturally instilled. This means that before children more fully learn what social behaviors are normative, social robots that exhibit abnormal behaviors, similar to people with ASD, are not seen as being different. This could imply that the sense of otherness attributed to people with ASD may not necessarily stem from some sort of natural innate discomfort, but rather be something learned that is driven by the additional costs of cognition associated with trying to understand behaviors perceived to be non-normative. Research also suggests that interacting with out-group members is inherently more cognitively taxing than interacting with in-group members [27], which may suggest that not knowing how to interact with an individual exhibiting behaviors associated with ASD could be more cognitively taxing, thereby leading to less empathy, and as a result, lower evaluations of warmth and competence. Thus, having an explanation for the behaviors by providing ASD diagnostic information may create a situation that is less cognitively taxing allowing for more empathy and in-group associations. This should in turn, allow for higher perceptions of warmth and competence. Thus, the third aim of the current study is to investigate if empathy towards an individual exhibiting behaviors associated with ASD mediates the relationship between providing ASD diagnostic information and perceptions of warmth and competence.

Overall, the current study had three major goals: (1) to extend the previous research to examine whether providing diagnostic information about an individual exhibiting behaviors associated with ASD would affect perceptions of competence and warmth, the two primary dimensions of social perception that have been argued to underlie stereotypes [10]; (2) by providing an explanation of the target having an ASD diagnosis versus not, this study hoped to find that having diagnostic information about the target could serve as an explanation for the exhibited ASD-related behaviors, creating a more fluent and less cognitively taxing situation, thereby increasing feelings of empathy [19]; and (3) to examine if empathy mediates the relationship between providing ASD diagnostic information and perceptions of warmth and competence, presumably because empathy should lead to higher in-group associations, which have been shown to be related to warmth and competence [25]. Therefore, it was predicted that participants would rate a target character who exhibited behaviors associated with ASD higher on competence and warmth if they knew the target had ASD compared to if they were not given any explanation for the displayed behaviors. It was also predicted that participants would feel more empathy towards the target if they were told the target had ASD compared to having no explanation for the behaviors displayed. Furthermore, if a providing diagnostic information about a target displaying ASD-like behaviors increases fluency and lessens the cognitive effort required in the situation, then providing diagnostic information should lead to greater empathy, which in turn should lead to higher ratings of warmth and competence.

## 2. Materials and Methods

### 2.1. Participants

A convenience sample of students at a small liberal arts institution was recruited to participate in an online study in exchange for either research experience credit in a participating psychology course or entry into a gift card lottery. A total of 90 individuals participated, of which 82 were considered for analysis. Participants were excluded from analysis if they scored less than 82% on the attention check or if they failed to correctly answer the manipulation check (*n* = 8). Three participants were excluded from the ASD Indicated Prime condition and five were excluded from the ASD Non-Indicated Prime condition. Participants ranged in age from 18–53 years old, with 85.3% of participants being of typical college age (18–22 years of age; *M* = 20.91). The majority of participants were Female (94%) and White (87.8%), with the remaining participants identifying as African or African American (9.7%), Indigenous American (4.9%), Asian or Asian American (2.4%), or chose not to specify (2.4%).

### 2.2. Instruments

#### 2.2.1. ASD Prime

The prime consisted of two, separate, randomly assigned introductions (ASD Indicated or ASD Non-Indicated, coded as 1 and 2, respectively) that were given before participants read a narrative about an interaction with a target character exhibiting behaviors associated with ASD. This introduction told participants that they would read a dialogue between Alex and Charlie, describing Alex as an employee at a grocery store (the target character) and Charlie as a customer looking for assistance (the interaction character). In the ASD Indicated Prime, participants were also told that Alex had been diagnosed with ASD, along with a brief explanation of the disorder. In the ASD Non-Indicated Prime, no additional information was given. Participants were then told to pay close attention to the narrative, as questions would be asked afterwards; this served as an attention check. No gender specific pronouns or physical descriptions were given to the characters, and the names Alex and Charlie were chosen because they are somewhat gender neutral and relatively common among several ethnicities.

#### 2.2.2. Social Competence

The Social Competence Inventory (Instrument S1) was used to assess how participants viewed the competency of the target character in the dialogue [31]. The scale consists of 17 statements (α = 0.747) which were modified to ask directly about the target character, named Alex. Participants used a five-point Likert scale to state how much they believed the statements applied to the target, such as “Alex can both give and take in social interactions”.

#### 2.2.3. Warmth

The Warmth-Related Behaviors Test (Instrument S2) was used to assess how participants viewed the friendliness and warmth of the target character in the dialogue [32]. The scale consists of 12 statements (α = 0.824) which were modified to ask directly about the target character. Participants used a five-point Likert scale to state how much they believed the statements applied to the target, such as “Alex thinks that people are fundamentally well-intentioned”.

#### 2.2.4. Empathy

Participants were asked to answer four questions (α = 0.786) created specifically for this study to assess empathy towards the target character exhibiting behaviors associated with ASD (Instrument S3). Participants were given statements about how they felt towards the target character and were asked on a five-point Likert scale to state how much they agreed with statements, such as “I believe I empathize more with Alex than Charlie in this scenario”.

#### 2.2.5. Attention Check

At the end of the study participants were asked seven questions about the details of the narrative they had read. These questions were presented with four multiple-choice answers, only one of which was correct (e.g., “What was Alex doing before Charlie approached?” and “When discussing pastries, Alex has what question about éclairs?”). These were included to confirm the participant had read the narrative closely and thus properly observed the ASD-related behaviors that were described.

#### 2.2.6. Manipulation Check

For the ASD Indicated Prime, an additional question was given to confirm that the prime was properly recognized. This manipulation check question asked, “Alex has what mental disorder?” and provided four options to choose from, consisting of ASD and three other mental disorders. No such question was asked if the participant was given the ASD Non-Indicated Prime.

#### 2.2.7. Demographics & Potential Covariates

Participants were asked a series of questions about demographic data and potential covariates. The demographic data asked the participants’ age, gender, sexual orientation, race, and ethnicity. The potential covariates included if participants knew someone who had, or if they themselves had ASD or other similar disorders (e.g., “Have you, a close friend, family member, or loved one been diagnosed with the following?”). Additionally, participants were asked about having job experiences similar to the target (e.g., “Have you ever worked in retail, or another similar job to the one Alex had?”). These potential covariates were designed to remove the influences of any previous experience that the participant may have had that might change their perceptions. However, it was found that none of these potential covariates reliably related to the dependent variables, and as such they were not included in the final analyses.

### 2.3. Procedures

This study consisted of an experimental design, with the ASD Prime as the independent variable, perceptions of social competence and warmth as the dependent variables, and empathy towards the target character as a mediating variable. After signing up for the study, individuals were given a link to the online study. They were first presented with informed consent information, and if they provided their consent, they were brought to the study materials.

Participants were randomly assigned to one of two conditions, which introduced the ASD Prime in the form of an introduction to a narrative about a social interaction between a grocery store clerk and a customer. The first group was given an introduction simply describing Alex (the target character) as a worker at a grocery store who is being approached by Charlie (the interaction character) for assistance (ASD Non-Indicated Prime; *n* = 47), whereas the other group was also told that Alex was diagnosed with ASD and given a brief explanation of the diagnosis (ASD Indicated Prime; *n* = 35). All participants were then asked to read the same short dialogue between the two characters, during which the target character displayed a number of behaviors associated with ASD (e.g., lack of eye contact, lack of understanding social cues, etc.). Participants were also told they would be asked questions about the narrative later. After participants read the narrative, they were asked to indicate their perceptions of the target. Specifically, they were asked questions about how warm and socially competent the target seemed. Additionally, participants were asked questions related to how empathetic they felt towards the target. After indicating their perceptions, participants answered questions to test their recollection of the material as well as a manipulation check. Lastly, participants were presented with questions related to demographic and covariate measures.

### 2.4. Data Analysis

Between-subjects *t*-tests were run using IBM SPSS Statistical Analysis software to determine if the ASD prime (ASD Non-Indicated Prime vs. ASD Indicated Prime) significantly affected how participants viewed the social competence and warmth of the target character as well as how empathetic they felt towards the target character. Additionally, a simple mediation analysis was performed using PROCESS to examine if empathy with the target mediated the relationship between ASD Prime and a composite variable of warmth and competence perceptions. The indirect effect was tested using a percentile bootstrap estimation approach with 5000 samples. The composite variable was calculated as a combination of the two individual rating scales to give a more complete picture of participants’ attitudes towards the target (α = 0.928). Z scores were first calculated individually for both target ratings and then averaged together to create the composite. The two components of the composite were significantly correlated to each other (*r* = 0.639, *p* < 0.001), and the same pattern of results emerged, such that participants given the ASD Indicated Prime rated the target higher on both rating scales compared to participants given the ASD Non-Indicated Prime. Furthermore, a factor analysis using a principal components analysis with direct oblimin rotation indicated that all items (social competence, 17; warmth 12) loaded onto Factor 1 that explained 29.37% of the variance in the data. Kaiser-Meyer-Olkin measure of sampling adequacy was 0.68, above the commonly recommended value of 0.6, and Bartlett’s test of sphericity was significant (χ^2^ (1225) = 2677.85, *p* < 0.001).

## 3. Results

### 3.1. Warmth and Competence Perceptions

**Figure 1:**
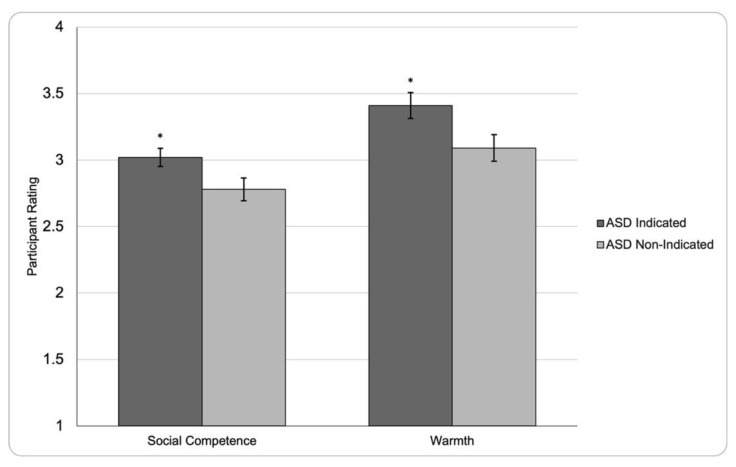
Perceptions of Social Competence and Warmth by ASD Prime. Mean participant rating of the target’s social competence and warmth on a scale from one to five, grouped by ASD Prime. Higher participant ratings represent greater perceived warmth and competence of the target. * represents *p* < 0.05 when compared to ASD Non-Indicated Prime. Vertical bars indicate error bars.

Between-subjects, *t*-tests were run to determine if the ASD Prime (ASD Non-Indicated Prime vs. ASD Indicated Prime) significantly affected how the participant viewed the target’s social competence and warmth. The results indicated that the ASD Prime did significantly affect how the participant viewed the target’s level of social competence, *t*(78) = 2.10, *p* = 0.039, Cohen’s* d* = 0.473. Specifically, it was found that participants given the ASD Indicated Prime (*M* = 3.02, *SD* = 0.40) rated the target as significantly more competent than participants given the ASD Non-Indicated Prime (*M* = 2.78, *SD* = 0.57). Similarly, results indicated that the ASD Prime significantly affected how warm the target was rated by the participants, *t*(79) = 2.15, *p* = 0.035, Cohen’s* d* = 0.484. Specifically, it was found that participants given the ASD Indicated Prime (*M* = 3.41, *SD* = 0.57) rated the target as exhibiting more warmth-related behaviors than participants given the ASD Non-Indicated Prime (*M* = 3.09, *SD* = 0.69). (See Figure 1).

### 3.2. Feelings of Empathy

**Figure 2:**
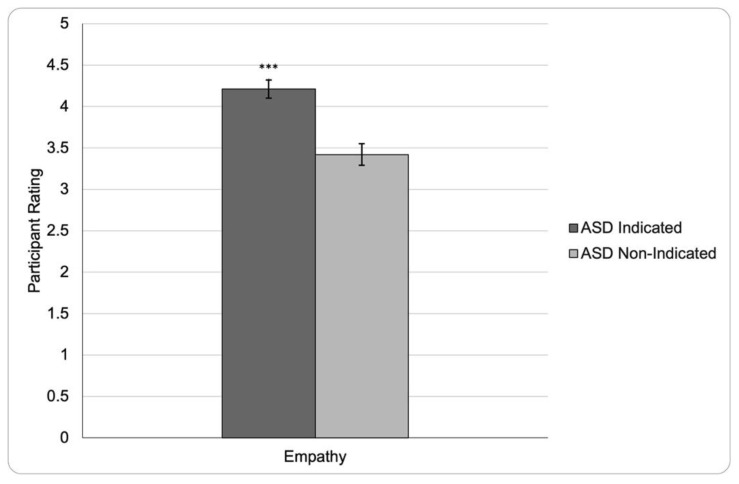
Feelings of Empathy by ASD Prime. Mean participant empathy felt towards the target character on a scale from one to five, grouped by ASD Prime. Higher participant ratings represent more empathy. *** represents *p* < 0.001 when compared to ASD Non-Indicated Prime. Vertical bars indicate error bars.

An additional between-subjects, *t*-test was run to determine if the ASD Prime (ASD Non-Indicated Prime vs. ASD Indicated Prime) significantly affected empathy felt towards the target. It was found that the ASD Prime did significantly affect how much empathy participants reported towards the target, *t*(80) = 4.38, *p* < 0.001, Cohen’s* d* = 0.979. Specifically, participants given the ASD Indicated Prime (*M* = 4.21, *SD* = 0.65) reported feeling more empathy towards the target than participants given the ASD Non-Indicated Prime (*M* = 3.42, *SD* = 0.90). (See Figure 2).

### 3.3. Mediation between ASD Prime and Ratings of Warmth and Competence

**Figure 3:**
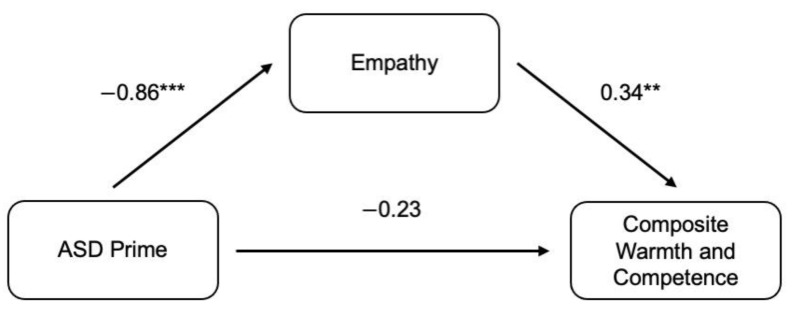
Empathy as a Mediator Between ASD Prime and Overall Target Rating. Mediation model of the indirect effects of ASD Prime on ratings of warmth and competence. ** represents *p* < 0.01, and *** represents *p* < 0.001.

A simple mediation analysis was performed using PROCESS to examine if empathy mediated the relationship between ASD Prime and ratings of warmth and competence. The ASD Indicated Prime predicted greater empathy (*B* = −0.78, *SE* = 0.19, 95% Confidence Interval (CI) [−1.15, −0.411]), β = −0.87, *p* < 0.001), and greater empathy was associated with higher composite warmth and competence ratings of the target, (*B* = 0.34, *SE* = 0.12, 95% CI [0.111, 0.586], β = 0.34, *p* < 0.005). The indirect effect was tested using a percentile bootstrap estimation approach with 5000 samples. (Effect = −0.272, 95% CI [−0.555, −0.047]). The direct effect of ASD Prime was no longer significant after controlling for the mediator (*B* = −0.21, *SE* = 0.22, 95% CI [−0.641, 0.220], β = −0.23, *p* = 0.332), consistent with full mediation. These results suggest that empathy mediated the indirect effect of ASD prime on ratings of warmth and competence. Specifically, the ASD Indicated Prime predicted more empathy, which in turn, predicted higher composite ratings of warmth and competence. (See Figure 3).

## 4. Discussion

The current study was designed to extend the previous research to examine whether providing diagnostic information about an individual exhibiting behaviors associated with ASD would affect perceptions of the individual, particularly related to the two primary dimensions of social perception (i.e., social competence and warmth). Furthermore, by providing an explanation of the target having an ASD diagnosis versus not, this study hoped to find that having diagnostic information about the target could serve as an explanation for the exhibited ASD-related behaviors, creating a more fluent and less cognitively taxing situation, thereby increasing feelings of empathy. Additionally, if empathy can lead to higher in-group associations, which have been shown to be related to warmth and competence, then the current study hoped to demonstrate that empathy mediates the relationship between providing ASD diagnostic information and perceptions of warmth and competence.

The results supported hypotheses. When given ASD diagnostic information, participants rated the target significantly higher in both warmth and competence, which represent the two primary dimensions of social perception [10,11,12,13]. Additionally, it was found that participants reported feeling significantly more empathy towards the target when they were told that the target had ASD and thus had a reason for the exhibited behaviors. Given that the target’s behaviors were held constant in both conditions, these results seem to suggest that being able to understand a reason for the target’s behavior may have lessened how cognitively taxing the situation was, allowing for more empathy towards the target and resulting judgments of warmth and competence. Indeed, greater feelings of empathy, in turn, predicted higher perceptions of warmth and competence. In other words, empathy with the target mediated the relationship between being given information about an ASD diagnosis and social perceptions of a person exhibiting behaviors associated with ASD.

Overall, the results of the current study are consistent with previous research. Specifically, Nah and Tan (2021) found that participants who were made aware of a student’s ASD diagnosis rated the student’s behaviors more positively compared to when they were not made aware of the diagnosis [16]. Brosnan and Mills (2016) indicated that participants’ affective responses were more positive after reading about a target displaying behaviors associated with ASD when the target was presented as someone diagnosed with ASD compared to a typical college student, and Matthews and colleagues (2015) presented vignettes of individuals exhibiting behaviors associated with ASD and found that a diagnosis of Level 1 ASD resulted in more positive behavioral and cognitive responses compared to those without a diagnosis [8,14]. Results of the current study also indicated that more positive responses occurred after being given diagnostic information, providing an explanation for the ASD-related behaviors exhibited in the vignette. However, instead of investigating participants’ own affective, cognitive, and behavioral responses, the current study examined perceptions directed more towards the target individual. Specifically, the current study is the first to examine perceptions of warmth and social competence perceived in an individual exhibiting behaviors associated with ASD. These two dimensions are important to investigate in relation to perceptions of others because they are the two primary dimensions of social perception on which humans are evolutionarily predisposed to judge others and have been shown to underlie stereotypes [10,11,12,13]. Thus including them in the current study expands the literature and provides important additional knowledge regarding how behaviors associated with ASD are perceived.

Furthermore, the results of the current study are consistent with previous research related to the idea of fluency and cognitive effort, such that, similar to Cameron and colleagues (2019), a presumably higher-efficacy situation (i.e., being given ASD diagnostic information and a reason for the behaviors) led to greater feelings of empathy [19]. Additionally, given that empathy has been argued to allow for more in-group associations [26], which are, in turn, related to perceptions of warmth and competence [10,25], the current finding that empathy mediates the relationship between ASD diagnostic information and perceptions of warmth and competence supports these ideas. Therefore, this is one of the first studies to provide evidence for a reason why information about an ASD diagnosis leads to more favorable ratings of someone exhibiting associated behaviors.

### 4.1. Implications

Overall, the findings of the current study could have important implications for the benefits of ASD awareness and understanding. Being given ASD diagnostic information and a brief explanation of the disorder led to greater feelings of empathy and more favorable ratings of warmth and competence. The current findings may also suggest that behaviors associated with ASD themselves do not seem to be what causes a social disconnect, but rather a lack of understanding of why the atypical behaviors are being displayed, which may make the situation more cognitively taxing. The brief explanation for the atypical behaviors displayed in the scenarios was enough to increase empathy and change how the exhibited ASD-related behaviors were perceived. This suggests that knowing more about ASD and understanding the behaviors commonly exhibited by people with ASD could help promote more positive perceptions during social interactions. Furthermore, if greater empathy allows for higher ratings of warmth and competence as shown in the current results, and warmth and competence underlie stereotypes [10,25], then the current study may provide evidence that disclosing one’s ASD diagnosis may help to reduce stereotypes associated with ASD-like behaviors. Information campaigns about conditions like ASD, such as neurodiversity training, could be effective in helping others to understand more about the diagnosis and facilitate smoother social interactions. The more someone knows about ASD, the more likely they are to pick up on specific behaviors and traits and understand why someone with ASD is exhibiting certain behaviors. These results may also explain why people with ASD tend to have fewer social relationships than those without, but are still able to maintain a few closer relationships, presumably because those closer relationships may be aware of the individual’s diagnosis. With a greater understanding of why their friend may behave in certain ways, their opinion should be more favorable. Overall, the results of the current study may indicate that people with ASD may want to consider disclosing their condition, rather than trying to hide it. Although many with ASD hide their diagnosis out of fear of being treated differently or being ostracized, having this explanation for their behavior may help someone with ASD find more acceptance in social situations. However, this decision should be considered along with other issues that could accompany the disclosure, such as prejudice, differential treatment, reduced privacy, bullying, and many others. Although the decision to disclose could provide some benefits consistent with the current study, this decision should remain in the hands of those with ASD to individually decide if self-disclosing would be beneficial for them.

### 4.2. Alternative Explanations

There are some alternative explanations for the findings in the current study. It is possible that participants who were given the ASD Indicated Prime rated the target more favorably not because of how they actually viewed the target, but because they felt pressured to do so. This may come from a social desirability bias, where participants may have worried how they would be viewed if they rated someone with ASD poorly, or from a desire to self-affirm they are a good person who is not prejudiced against someone with an ASD diagnosis. However, anonymity was prioritized in the study, which may have helped mitigate the impact of a potential social desirability bias.

Additionally, it may have been the case that because the ASD Indicated Prime provided participants with an additional personal detail about the target, but the ASD Non-Indicated Prime did not, participants in the ASD Indicated Prime group may have rated the target more favorably simply because they knew additional information about them. However, results did indicate that prior experience with ASD predicted more favorable ratings of the target regardless of condition, which provides support for the idea that greater understanding of the behaviors could be what is driving the effects rather than an additional personal detail.

### 4.3. Limitations

The sample used for this study consisted of a convenience sample, composed of traditionally college-aged students, which may have potentially influenced the results. Specifically, younger generations and more educated individuals may be more familiar with mental health issues and ASD in particular, and thus more understanding of the related behaviors. Consequently, the results may change if older participants were to be used in replications. The participants were also female by a large majority. Research suggests that females have greater empathetic responses than males and that these differences may grow with age [33]. In fact, one study indicated that females were more likely to rate those with ASD more favorably than males [17]. Therefore, if more males were included in the sample, it is possible that interaction effects would exist, such that the difference between groups would be greater for females, but perhaps not for males. Finally, a majority of participants in this study were Americans who were White. In the United States, the rates of ASD diagnosis are relatively equal across racial boundaries [34]. However, there could be other racial and cultural differences that would influence how these individuals would respond to the target. For example, those from more collectivist cultures have been shown to exhibit greater prejudice towards out-group members and may therefore view the target more harshly when aware of the ASD diagnosis [35]. Although neither age, gender, nor race predicted empathy or target ratings in the current study, the limited variability of these demographics in the sample may not have granted enough power to find such effects. Thus, future research would benefit from a more diverse sample.

### 4.4. Future Research

Future research should take steps to help rule out alternative explanations. For example, it may be wise to give the ASD Non-Indicated group a piece of personal information about the target, unrelated to the target’s behavior, in order to rule out the possibility that the mere exposure of additional information was the determining factor. Another possibility would be to implicitly prime participants with the explanation of the target’s behavior instead of explicitly stating the diagnosis. This way the participants could still make the cognitive association between the behaviors shown and the explanation of an ASD diagnosis, but they would not be as influenced by social desirability factors. Additionally, the current study examined explicit perceptions of behaviors associated with ASD. Previous research suggests that implicit attitudes towards those with ASD are less flexible than explicit and that explicit attitudes are more likely to improve with exposure to ASD [36]. It would be helpful to see if providing diagnostic information would lead to more favorable implicit perceptions of ASD-like behaviors in addition to the explicit perceptions shown in the current study.

Future research could also include additional primes to see if this pattern of results is unique to ASD or if it can be applied to other potential explanations for the target’s behavior. Explanations such as the target having ADHD, schizophrenia, or having a history of substance addiction could be examined to see if they differ from when a participant is told the target has ASD. Depending on the results, this could suggest that having empathy associated with the explanation is an important factor, rather than just having any explanation at all. Additionally, further studies could include participants who have an ASD diagnoses themselves to see if they would view the target differently given that they may have the greatest level of understanding for the exhibited behaviors. Previous research has found that those with ASD did not view social robots negatively based upon their perceived social deficits [29], and that those with ASD were found to hold less stereotypical attitudes about others in general than those without ASD [18]. These results could imply that individuals with ASD would rate the target equivalently and not need an additional explanation for the behaviors because they already understand them. Additionally, compared to others without ASD, those with the diagnosis, due to their personal experience with associated behaviors, may empathize even more with the target and rate the target even more favorably when given the ASD diagnostic information. Lastly, future research could confirm that the mechanism that links empathy to higher ratings of warmth and competence is indeed the ability to make greater in-group associations. The current study only provides theoretical evidence of such mechanism.

## 5. Conclusions

In conclusion, the current study found that people tended to view someone who exhibited behaviors associated with ASD more positively, particularly on dimensions of warmth and competence after being given a diagnosis that could explain those behaviors and that empathy mediates this relationship. This has important implications going forward for how we approach education and increasing understanding about disorders such as ASD, as well as for making the decision whether or not to disclose one’s diagnosis. Additionally, this study also challenges the idea that behaviors associated with ASD are inherently negative, instead suggesting that they are looked down upon because they differ from what is expected, making the situation more cognitively taxing. Finally, the results of this study further the understanding of how people view each other, specifically on the two fundamental traits of warmth and social competence.

## Data Availability

The data presented in this study are available from the corresponding author upon request.

## Supplementary Materials

The following supporting information can be downloaded at: https://www.mdpi.com/article/10.3390/ejihpe12110112/s1, Instrument S1: Social Competence Inventory; Instrument S2: Warmth-Related Behaviors Test; Instrument S3: Empathy.
