# Supplementary material for: Perceptions of Behaviors Associated with ASD in Others: Knowledge of the Diagnosis Increases Empathy and Improves Perceptions of Warmth and Competence
Source: Eur J Investig Health Psychol Educ. 2022 Nov 4;12(11):1594–606. doi: 10.3390/ejihpe12110112 (PMC9689353; doi:10.3390/ejihpe12110112)
Supplement: Supplementary file 1 [file ejihpe-12-00112-s001.zip › ejihpe-1846894-supplementary.pdf]

Materials and Methods (Instruments)

Instrument S1 – Social Competence Inventory

Alex is helpful to customers
Alex is likely to help a coworker who is sick
Alex is likely to encourage shy people into conversation
Alex is able to empathize with strangers
Alex is good at preventing conflict
Alex is hesitant to interact with strangers
Alex is able to feel joy when others are happy
Alex lets others dominate the conversation
Alex can both give and take in social interactions
Alex helps others clean after themself
Alex often criticizes peers
Alex often suggests activities to others
Alex gets along well with coworkers
Alex gets many complaints from customers
Alex compliments coworkers
Alex often acts socially withdrawn during staff meetings
Alex tends to find solutions when in conflict with others

Instrument S2 – Warmth-Related Behaviors Test

Alex is likely to smile at strangers on the street to make their day more pleasant.
If asked, Alex would wake up at 5:00 in the morning to drive a friend to the airport.
Alex would bring some fruits to work to share with colleagues.
Alex is someone who easily makes new friends.
Alex is the kind of person who cares about others well-being.
Alex thinks that people are fundamentally well-intentioned.
Alex is not always available for others even if they need help.
Alex would leave a party thinking that people there were too superficial.
Alex does not really care about what others are doing.
Alex sometimes says irritating or even rude things to other people.
Alex would not like it if a passerby asked for directions.
Alex is the kind of person who thinks they have no business in others' misfortune.

Instrument S3 – Empathy
I believe I empathize with Alex more than Charlie after reading the scenario
Being mean to Alex would be an upsetting thought for me
The thought of Alex being fired for this encounter upsets me
I feel distress over bad things happening to Alex
